# The relationship between chronic PTSD, cortical volumetry and white matter microstructure among Australian combat veterans

**DOI:** 10.1186/s40779-022-00413-z

**Published:** 2022-09-16

**Authors:** Madeline Romaniuk, Ying Xia, Gina Fisher, Kerstin Pannek, Jurgen Fripp, Justine Evans, Stephen Rose

**Affiliations:** 1 Gallipoli Medical Research Foundation, Greenslopes Private Hospital, Greenslopes, 4120 Australia; 2 Faculty of Health and Behavioural Sciences, The University of Queensland, Saint Lucia, 4067 Australia; 3 The Australian E-Health Research Centre, CSIRO Health and Biosecurity, Herston, 4029 Australia

**Keywords:** Posttraumatic stress disorder (PTSD), Veterans, Diffusion tensor imaging, Magnetic resonance imaging (MRI), Fractional anisotropy

## Abstract

**Background:**

Posttraumatic stress disorder (PTSD) has been associated with volumetric and white matter microstructural changes among general and veteran populations. However, regions implicated have greatly varied and often conflict between studies, potentially due to confounding comorbidities within samples. This study compared grey matter volume and white matter microstructure among Australian combat veterans with and without a lifetime diagnosis of PTSD, in a homogenous sample assessed for known confounding comorbidities.

**Methods:**

Sixty-eight male trauma-exposed veterans (16 PTSD-diagnosed; mean age 69 years) completed a battery of psychometric assessments and underwent magnetic resonance and diffusion tensor imaging. Analyses included tract-based spatial statistics, voxel-wise analyses, diffusion connectome-based group-wise analysis, and volumetric analysis.

**Results:**

Significantly smaller grey matter volumes were observed in the left prefrontal cortex (*P* = 0.026), bilateral middle frontal gyrus (*P* = 0.021), and left anterior insula (*P* = 0.048) in the PTSD group compared to controls. Significant negative correlations were found between PTSD symptom severity and fractional anisotropy values in the left corticospinal tract (*R*^*2*^ = 0.34, *P* = 0.024) and left inferior cerebellar peduncle (*R*^2^ = 0.62, *P* = 0.016). No connectome-based differences in white matter properties were observed.

**Conclusions:**

Findings from this study reinforce reports of white matter alterations, as indicated by reduced fractional anisotropy values, in relation to PTSD symptom severity, as well as patterns of reduced volume in the prefrontal cortex. These results contribute to the developing profile of neuroanatomical differences uniquely attributable to veterans who suffer from chronic PTSD.

## Background

Posttraumatic stress disorder (PTSD) is a debilitating psychiatric condition precipitated by exposure to a traumatic event [1]. In military veteran populations, PTSD prevalence estimates are almost double that in the general population (21.4%) [2–4], reflecting a higher risk of exposure to traumatic events due to military service and combat deployments. Chronic PTSD has been defined as experiencing symptom duration of more than 3 months [1]. In a longitudinal study of Australian Vietnam veterans (*n* = 388), 50.3% of PTSD sufferers had a current PTSD diagnosis 14 years following initial assessment, indicating chronic PTSD is a concern among this cohort of veterans [3, 4].

In addition to the severe functional impact PTSD can have, it has been established that biological changes are also associated with the condition, including brain atrophy [5]. A large body of research has aimed to identify specific regions that demonstrate significant volume reduction in individuals with PTSD. Commonly reported regions of grey matter (GM) atrophy in those with PTSD include the hippocampus, a region governing processes involved in memory, the anterior cingulate cortex (ACC), involved in emotion regulation, and the prefrontal cortex (PFC), implicated in emotion and attention regulation [5–7]. Reduced volume has also been documented in the amygdala [5], a region of the brain implicated in fear processing and threat appraisal [7], although discrepant findings have been observed in adult-onset PTSD studies [8].

More recently, functional changes in white matter (WM) neuronal networks have been assessed using Diffusion Tensor Imaging (DTI), which determines the diffusion and directionality of water molecules [9]. A key metric in DTI is fractional anisotropy (FA), a scalar value between zero and one that describes the degree of directional restriction of the diffusion of water. It is considered a global index of WM microstructure that encompasses fibre orientation, myelination, and axonal density [9]. DTI metrics also include mean diffusivity (MD) which is an inverse measure of membrane density [9]. WM plasticity refers to the propensity for neuronal networks to change in response to learning or maturation [10]. Indices of WM microstructure have been observed to correlate with PTSD symptom severity [11, 12], however, there is little consensus regarding the particular regions of WM implicated, as well as the directionality of findings.

A number of meta-analyses have assessed findings from DTI studies that compared WM microstructure between PTSD and trauma-exposed, or non-trauma exposed healthy control groups in samples with varied trauma backgrounds [10, 13–16]. The cingulum bundle has been a relatively robust region of WM microstructural differences in PTSD-diagnosed groups, however, numerous regions have also exhibited conflicting outcomes of both increased and decreased FA [10, 13–16]. In line with the most consistent areas of WM differences (cingulum, frontal WM tracts, and superior longitudinal fasciculus) it has been hypothesised that alterations may be reflective of differences in emotional processing, the extinction of aversive memories, and context learning related to PTSD symptoms [10]. Differences in WM in these areas may be the result of exposure to trauma, symptoms of PTSD, or may be pre-existing differences in these samples constituting a predisposing factor [10]. The neurobiological model of PTSD posits that abnormalities in regions associated with fear learning, threat detection, emotion regulation, and contextual processing can perpetuate PTSD symptoms such as persistent fear responses (i.e., avoidance) and mood symptoms (i.e., anger and irritability) [2].

The heterogeneity of participant samples is a limitation of extant research that may be responsible for the lack of consistent findings [13, 15]. Study samples frequently involve a large range in age and PTSD chronicity, which may influence the degree of neuroanatomical changes detected [11, 12, 17–19]. In addition to age and symptom chronicity, the importance of accounting for comorbid conditions such as depression has also been highlighted in recent reviews, as such symptoms are associated with WM differences [10, 14, 15]. Finally, it has been argued that differences in the nature, frequency, and duration of trauma exposure may result in differential findings [10, 13, 20]. Reuveni and colleagues [20] noted that trauma type (civilian versus combat-related), and number of years since trauma modulated patterns of WM differences observed, while a recent review also highlighted differential WM patterns when grouping participants based on childhood versus adult trauma [10].

Few studies have focused specifically on combat veterans and PTSD-related WM abnormalities, with the majority performed on relatively young cohorts presenting with predominantly acute PTSD presentations. Studies have included veterans from the U.S. military [17, 21–23], mixed service backgrounds [18], the Netherlands [11, 24] and the Israeli Defence Force [20]. In veteran studies, the cingulum has been the most commonly reported region of WM abnormality, however, the direction of FA findings, as well as other regions implicated, have widely varied.

Previous veteran DTI studies appear to be limited by inconsistent inclusion/exclusion strategies. Factors reported to be associated with cortical changes, including traumatic brain injury (TBI) [22], major depressive disorder (MDD) [15], alcohol dependence [25], and antidepressant medications [26] have typically not been screened or reported within samples, or statistically controlled for within the cohort. Further, some studies have not included a combat-trauma-exposed control (non-PTSD diagnosed) group. This poses limitations in interpreting the unique impact of chronic PTSD symptoms on neuroanatomical structures, beyond changes that may be attributable to trauma exposure alone [27]. The lack of DTI studies that clearly describe or limit these potential confounding factors might be contributing to the heterogeneity of findings to date.

Achieving a more thorough classification of biological mechanisms or abnormalities in chronic PTSD may be critical for differentiating the stages of the disorder (i.e., acute versus chronic presentations) in a way that enables more sensitive application of appropriate therapeutic approaches [28]. It is important to gain an understanding of the way chronic PTSD affects the neuroanatomical structure of veterans who comprise a substantial proportion of PTSD-diagnosed individuals, particularly since more enduring deterioration of functional areas may be present, and considering the degree to which neuroplasticity is correlated with age. This has clinical implications for understanding potential impacts of neuroanatomical changes on the treatment response and likelihood of remission, in light of emerging research assessing longitudinal changes in brain structures following engagement in evidence-based treatments such as trauma-focused cognitive behaviour therapy (TF CBT) [29].

This study aimed to examine a sample free of potentially confounding medical and psychiatric factors [traumatic brain injury (TBI), MDD, alcohol dependence, and current antidepressant medication use] in order to: 1) compare cortical and deep GM volumes between PTSD and trauma-exposed control groups, as well as determine the relationship between PTSD symptom severity and cortical volume, 2) compare diffusion tensor measures of FA and MD between PTSD and trauma-exposed control groups to determine the relationship between PTSD symptom severity and FA and MD, and 3) compare WM tracts between PTSD and trauma-exposed control groups using connectome-based group-wise analysis. The a-priori regions of interest for volume analysis included the amygdala, hippocampus, ACC, and PFC, in line with literature. Specific hypotheses were not proposed due to the variation among samples and findings to date.

## Method

### Participants

Participants included 68 combat-exposed, former members of the Australian Defence Force (ADF) who served in the Vietnam War, aged 64 to 88 years [mean (M) = 69.0, standard deviation (SD) = 4.0]. Participants were grouped into lifetime chronic PTSD (*n* = 16) and trauma-exposed control (*n* = 52) conditions using the Clinician-Administered PTSD Scale for Diagnostic and Statistical Manual of Mental Disorders (DSM-5) (CAPS-5), which provided both diagnostic lifetime status and current symptom severity [30]. PTSD participants and trauma-exposed controls were not matched, however, all were male and groups did not significantly differ in age or years of military service. PTSD and control participants were excluded if they: 1) had a current diagnosis of MDD, Bipolar Disorder, Alcohol Dependence, Schizophrenia, or current psychosis; 2) had a history of TBI, 3) were currently taking antidepressant medications; or 4) were not exposed to trauma according to the DSM-5 Criterion A for PTSD. Participants in the trauma-exposed control condition did not meet criteria for either current or lifetime history of PTSD. Participants in the PTSD group had a considerable average symptom duration of 30.95 years (SD = 15.61), and were experiencing PTSD severity scores ranging from 7 to 35 (M = 19.81, SD = 7.94) at the time of the assessment. Participants were recruited via a specialized veteran mental health unit at Greenslopes Private Hospital (GPH) in Brisbane, Australia, as well as through the Gallipoli Medical Research Foundation (GMRF) and Returned and Services League (RSL) of Australia websites, RSL publications, newspaper and television advertisements, and by word of mouth.

### Ethics

The study protocol was approved by the Greenslopes Research and Ethics Committee, the Department of Veterans Affairs Human Research Ethics Committee, The University of Queensland Ethics Committee, and the Queensland University of Technology Human Research Ethics Committee. The study was registered with the Australian New Zealand Clinical Trials Registry (ACTRN12614000429651) and carried out in accordance with the National Statement on Ethical Conduct in Research Involving Humans (National Health and Medical Research Council of Australia, 2007). Participants were taken through an informed consent procedure before providing written informed consent for all medical and psychological assessments and test procedures.

### Assessments

Doctoral-level clinical psychologists administered structured diagnostic assessments. Psychiatric conditions were screened using the Mini International Neuropsychiatric Interview (MINI) [31] and the Alcohol Use Disorders Identification Test (AUDIT) [32]. Previous TBI was assessed by a medical officer for each participant. Participants were asked if they had “*ever had a significant head injury, or been diagnosed as having a mild TBI or concussion*”. If participants responded “yes”, or “unsure”, a series of questions pertaining to the nature of symptoms and the injury were asked, including whether and how long the participant lost consciousness, the specific symptoms they experienced at the time of the injury, how long symptoms persisted, what medical attention they received, and if they received medical imaging. Magnetic resonance imaging (MRI) scans were also reviewed by a radiologist for evidence of injury. Cognitive impairment was assessed with the Montreal Cognitive Assessment (MoCA) [33]. A 10-item military history questionnaire was also administered to record service details, and medical officers at GMRF performed a structured clinical history and examination.

### MRI data acquisition

MRI acquisition was performed using a Magnetom Verio 3 T scanner (Siemens, Erlangen, Germany). Table 1 presents acquisition parameters for MR sequences including T1-weighted magnetization-prepared rapid gradient-echo (MPRAGE), T2-weighted fluid-attenuated inversion recovery (FLAIR) and diffusion-weighted images along with a field map to aid correction of susceptibility distortions. All images were reviewed for neuroanatomical defects by qualified radiologists, who reported nil abnormal findings.

**Table 1:** Magnetic resonance imaging (MRI) parameters

Parameter	T1-weighted MPRAGE	T2-weighted FLAIR	Diffusion-weighted Images	Field map
Scan timing				
Echo time (TE, ms)	2.93	82	130	5.19 / 7.65
Repetition time (TR, ms)	2300	6000	8900	400
Inversion time (TI, ms)	900	2070	–	–
Flip angle (°)	9	125	90	60
Bandwidth (BW, Hz/pixel)	241	200	992	260
Echo train length (ETL)	1	24	1	1
b-values	–	–	0, 3000	–
Acquisition timing				
Frequency (number of pixels)	240	320	120	64
Phase (number of pixels)	256	320	120	64
Phase field of view (FOV, %)	0.94	1.00	1.00	100
Gradient directions	–	–	64	–
b-zeros	–	–	1	–
Scanning range				
FOV (mm)	253 × 270	250 × 250	240 × 240	192 × 192
Spacing (mm)	1.05	0.78	3	3.75
Slice thickness (mm)	1.2	2.5	2	3
Slices	192	60	50	36

*MPRAGE* magnetization-prepared rapid gradient-echo, *FLAIR* fluid-attenuated inversion recovery

### MRI data processing

The 3D T1-weighted MPRAGE images for all participants were first segmented into GM, WM, and cerebrospinal fluid (CSF) tissues using an in-house implementation of the expectation maximization algorithm of van Leemput and colleagues [34]. The T1-weighted data were further parcellated into cortical and sub-cortical regions (mainly GM) based on the automated anatomic labeling (AAL) and Neuromorphometrics parcellation atlases using Learning Embeddings for Atlas Propagation following the work of Wolz and colleagues [35]. This method relies on the construction of an affinity matrix that captures the similarity between each pair of images in the atlas database (M = 20 atlases used here), and therefore, minimizes the amount of deformation required for segmentation propagation. The groups did not significantly differ on WM hyperintensities (*P* < 0.05) automatically quantified from 2D FLAIR images [36].

**Fig. 1:**
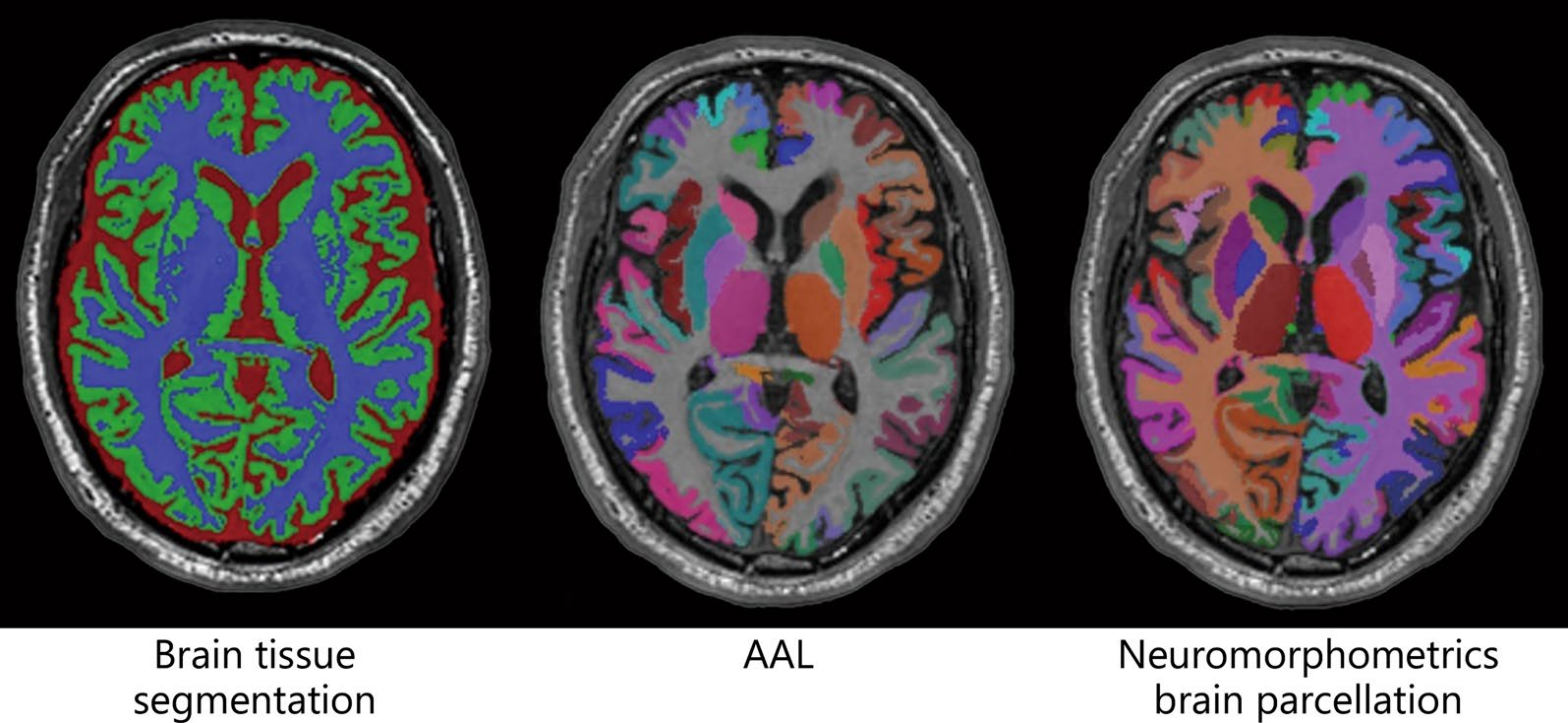
Axial cross-sections showing example brain segmentation and parcellation labels overlaid on the structural MRI. Brain tissue segmentation (left), coloured brain regions defined in the automated anatomical labeling (AAL) atlas (middle), coloured brain regions defined in the Neuromorphometrics atlas (right). AAL Automated anatomic labeling

The AAL brain parcellation was used in structural connectome generation for the diffusion data. A connectome is the complete map of the neural connections in a brain. The Neuromorphometric brain parcellation provided the regions of interest (ROI) for cortical volume analysis. To account for individual differences in head size, the volumetric measures were expressed as a percentage of total intracranial volume. Figure 1 shows example structural outputs from T1-weighted data including brain tissue segmentation and whole-brain parcellation.

Diffusion MR images were first corrected for image artefacts caused by involuntary head motion, cardiac pulsation, and image distortions. Volumes containing within-volume motion were automatically detected and removed from further analysis. Diffusion data were then corrected for head motion using Functional MRI of the Brain (FMRIB) software library (FSL) eddy [37], susceptibility distortions corrected using the field map, and intensity inhomogeneities using N4 [38]. FA and MD images were estimated from the corrected diffusion data using a diffusion tensor model.

Fiber-orientation distributions were estimated using MRtrix3 (MRtrix.org) [39], and whole-brain tractography was performed on the corrected diffusion data using MRtrix3 [39, 40]. Diffusion and T1-weighted structural images were firstly co-registered using boundary-based registration [41]. The resulting transformation was applied to the header information of structural images with no downsampling performed. A five-tissue type (WM, cortical GM, deep GM, CSF, other) mask was calculated from transformed structural images in the diffusion space. Anatomically constrained tractography [40] was used to generate 100 million probabilistic streamlines, which were subsequently filtered to 10 million streamlines using spherical-deconvolution informed filtering [42].

**Fig. 2:**
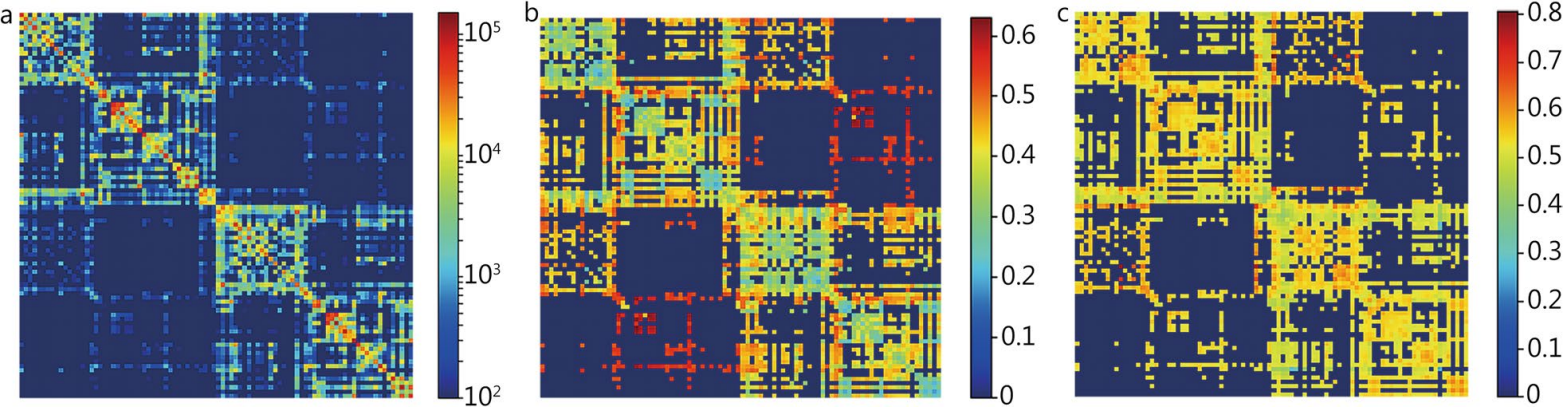
Average connectivity matrices from all subjects in the study that encode number of streamlines. **a** Number of streamlines. **b** Average FA values of connections. **c** Average MD values of connections. Connections with the streamline numbers < 100 were masked in the matrix of average FA values of connections and average MD values of connections. FA fractional anisotropy, MD mean diffusivity

The AAL parcellation obtained from T1-weighted data was transformed to the diffusion space (without downsampling), to parcellate the brain into 90 regions (excluding the cerebellum). Connectivity matrices of size 90 × 90 were generated by encoding the number of streamlines, average FA values of connections and average MD values of connections between each pair of regions in the AAL atlas. An average connectivity matrix of MNUM was calculated from the study cohort (Fig. 2) and connections with an average streamline number < 100 were removed from further analysis.

### DTI tract-based spatial statistics

**Fig. 3:**
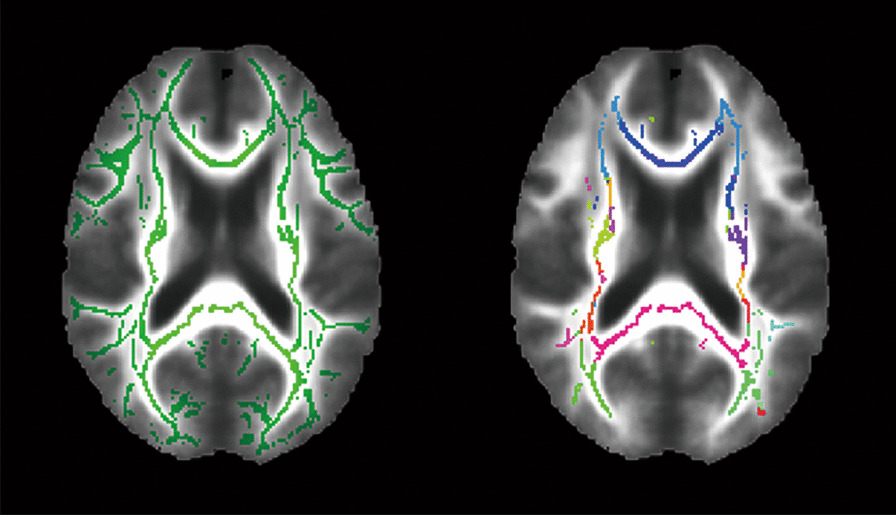
Mean FA skeleton (Green, left) across all subjects overlaid on the mean FA image used for the voxel-wise analysis and the coloured intersection regions between the mean FA skeleton and JHU white matter tractography atlas used for the region-of-interest based analysis (right). FA fractional anisotropy, JHU John Hopkins University

Tract-based spatial statistics (TBSS) [43] were performed as implemented in the FMRIB Software Library (FSL; version 6, Analysis Group, FMRIB, Oxford, UK). All FA images were aligned to standard space, and a mean FA skeleton mask was created using an FA threshold of 0.2 that was applied to the average FA skeleton in order to limit voxels to WM [43]. Figure 3 (left) shows the FA skeleton mask. Voxel-wise nonparametric statistical comparison between PTSD and control groups was performed using the “randomise” algorithm in FSL [37] with 5000 permutations. Corrections for multiple voxel-wise comparisons were performed using threshold-free cluster enhancement (TFCE) [44]. Age was used as the confounding variable. Voxel-wise statistical analyses were performed on both FA and MD images, which allows for the identification of anatomic locations of voxel clusters with statistically significant differences (*P* < 0.05, corrected for multiple comparisons) in the two diffusion tensor measures between groups. Corrections were not applied for multiple comparisons.

In addition to the whole brain voxel-wise approach, ROI analyses of FA and MD images were conducted within the intersection of the mean FA skeleton mask and John Hopkins University (JHU) atlas that parcellates the WM regions into 48 ROIs [45]. Figure 3 (right) shows the resulting regions. Average FA and MD within each of these ROIs were then determined for each participant. Correlations of FA and MD measures and CAPS-5 PTSD severity scores were investigated within each of the ROIs in the PTSD diagnosed participants.

### Diffusion connectome-based group-wise analysis

Connectivity matrices were contrasted for group-wise comparison using the Network-Based Statistics (NBS) toolbox [46], which identified differences in the number of streamlines or tract-averaged FA and MD values between groups for every connection. Age was used as the confounding variable. In connectome-based group-wise analyses, a *t*-test was used for the univariate testing at every connection in the network. A *t*-statistic threshold was chosen empirically to define a set of supra-threshold connections with *P* < 0.05. Any connected component defined by these supra-threshold connections was then identified. *P*-values for each of these components were corrected for the family-wise error (FWE) using permutation testing. In this analysis, a *t*-test with 5000 permutations was performed and tested a range of t-statistic thresholds (1.0–3.5) in order to determine the highest threshold value at which the number of significantly different connections plateaued.

### Volumetric analysis

All analyses were performed in the R environment (version 3.4.0, R Foundation for Statistical Computing, Vienna, Austria). The volumetric analysis was conducted firstly on the four prior ROIs (amygdala, hippocampus, ACC and PFC) and then on the brain regions in Neuromorphometrics parcellation for exploratory purposes. The distribution of the volumes was assessed for normality using the Shapiro–Wilk test. Multivariate analyses of variance (MANOVA) were conducted on volumetric measures of brain regions using a two-factor design, where group was the between-participant factor, hemisphere was the within-participant factor, and age was the covariate. To further examine the relationship between brain structures and PTSD symptom severity, partial correlations controlling for age were calculated between the analysed measures and CAPS-5 severity scores in the group of participants diagnosed with PTSD. A significance level of *P* < 0.05 was used for all analyses. No corrections were applied for unequal group sizes.

## Results

### Demographic and clinical data

Demographic and clinical data for the full sample are detailed in Table 2. All participants in the sample met criterion A for trauma exposure, as defined by DSM-5. Further, 87.50% of participants in the PTSD group and 88.46% of participants in the trauma-exposed control group had reportedly been exposed directly to a life threat. The remainder of the sample was either exposed to a life threat of another or a serious injury of another. No participant reported illicit drug use as assessed by the MINI.

**Table 2:** Descriptive statistics and mean comparisons for demographic and clinical characteristics between the PTSD-diagnosed and trauma-exposed control group

Characteristic	PTSD-diagnosed (*n* = 16)	Trauma-exposed non-PTSD diagnosed (*n* = 52)	*P-*value
Ethnicity [*n* (%)]			
Caucasian	12 (75.00)	49 (94.23)	–
Other	1 (6.25)	–	–
Not reported	3 (18.75)	3 (5.77)	–
Education level [*n* (%)]			
≤ Year 10	8 (50.00)	9 (17.31)	–
≤ Year 12	5 (31.25)	15 (28.85)	–
Vocational	-	7 (13.46)	–
University	3 (18.75)	21 (40.38)	–
Type of service [*n* (%)]			
Army	16 (100.0)	42 (80.77)	–
Navy	–	2 (3.85)	–
Air Force	–	8 (15.38)	–
Age (years, M ± SD)	69.75 ± 5.53	68.71 ± 3.43	0.368
Years of service (M ± SD)	13.60 ± 10.41^*^	17.03 ± 12.50	0.338
AUDIT total (M ± SD)	7.13 ± 5.24	6.35 ± 3.73	0.511
MoCA total (M ± SD)	25.94 ± 2.62	26.90 ± 2.04	0.127
CAPS PTSD symptom severity score (M ± SD)	19.81 ± 7.94	3.52 ± 4.35	< 0.001
PTSD duration in years (M ± SD)	30.95 ± 15.61	N/A	–

*PTSD* posttraumatic stress disorder, *M* mean, *SD* standard deviation

^*^n = 15, service year data missing for one participant in the PTSD-diagnosed group

### MR volumetric analysis

Significantly smaller volumes were observed in PTSD participants for the left PFC (*P* = 0.026), the middle frontal gyrus (i.e., the subregion of PFC) on both left and right (*P* = 0.011 and *P* = 0.017, respectively), and left anterior insula (*P* = 0.048). The PTSD group was found to have significantly larger volumes of the left anterior orbital gyrus (*P* = 0.016) than the control group. Table 3 details the results of MANOVA analyses in these brain regions. The correlation analyses in the PTSD group, controlling for age, revealed a negative correlation observed at the right central operculum [*F* (1, 13) = 6.186, *P* = 0.027], and a positive correlation at the right inferior occipital gyrus [*F* (1, 13) = 5.242, *P* = 0.039].

**Table 3:** Group comparisons of normalized brain region volumes (in %) between PTSD participants and controls

Brain region	Side	PTSD (*n* = 16)	Controls (*n* = 52)	*F*-value	*P*-value
Amygdala				1.529	–
	Left	0.075 ± 0.008	0.075 ± 0.007	0.041	–
	Right	0.078 ± 0.006	0.075 ± 0.009	1.184	–
Hippocampus				0.843	–
	Left	0.217 ± 0.023	0.215 ± 0.019	0.314	–
	Right	0.228 ± 0.021	0.221 ± 0.021	1.481	–
Anterior cingulate cortex (ACC)				0.560	–
	Left	0.261 ± 0.040	0.252 ± 0.047	0.709	–
	Right	0.225 ± 0.029	0.229 ± 0.042	0.135	–
Prefrontal cortex (PFC)				2.851	0.065 ^+^
	Left	2.239 ± 0.156	2.331 ± 0.122	5.226	0.026 ^*^
	Right	2.283 ± 0.144	2.353 ± 0.138	2.492	–
Middle frontal gyrus				4.126	0.021 ^*^
	Left	0.865 ± 0.073	0.923 ± 0.076	6.900	0.011 ^*^
	Right	0.871 ± 0.115	0.936 ± 0.081	6.030	0.017 ^*^
Anterior insula				2.492	0.091 ^+^
	Left	0.231 ± 0.025	0.244 ± 0.021	4.056	0.048 ^*^
	Right	0.237 ± 0.018	0.243 ± 0.022	0.914	–
Anterior orbital gyrus				3.035	0.055 ^+^
	Left	0.112 ± 0.016	0.100 ± 0.019	6.072	0.016 ^*^
	Right	0.118 ± 0.023	0.114 ± 0.014	1.082	–

The *P*-values have not been adjusted for multiple comparisons and were coded using the following significance codes: ^*^P < 0.05, ^+^*P* < 0.1, ^−^*P* < 1. *PTSD* posttraumatic stress disorder

### Tract-based spatial statistics (TBSS)

**Fig. 4:**
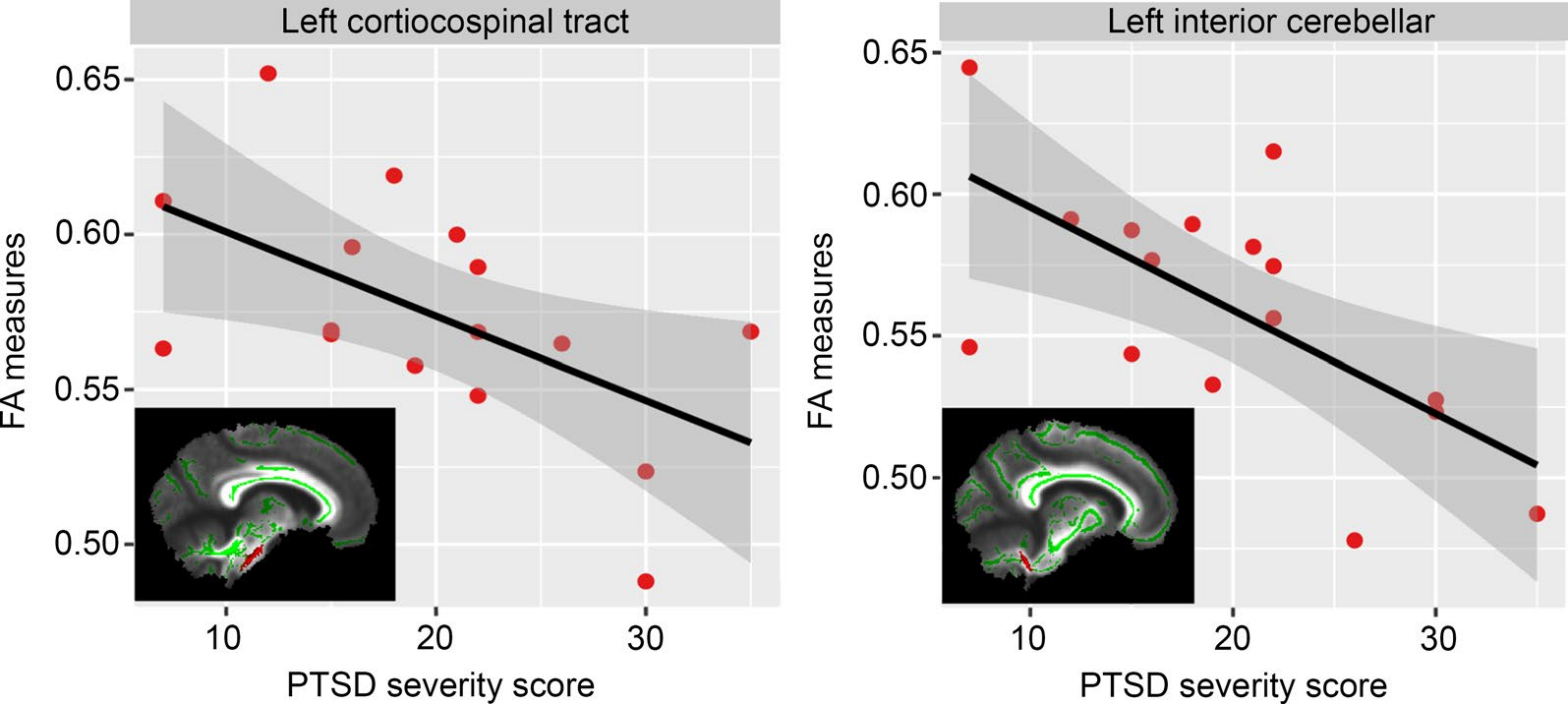
Correlation plots between regional FA measures and PTSD severity scores for PTSD participants. Significant negative correlation with PTSD severity scores were identified for left corticospinal tract (*R*^2^ = 0.34, left) and left inferior cerebellar (*R*^*2*^ = 0.62, right). These white matter tract regions were displayed in the bottom left corner and overlaid on the mean FA skeleton (Green). FA fractional anisotropy, PTSD posttraumatic stress disorder

No local area of the WM skeleton mask demonstrated statistically significant reduced FA or increased MD between PTSD participants and controls using TBSS and voxel-wise nonparametric statistical comparison. Analyses between PTSD severity scores and regional FA values in the PTSD group showed significant negative correlations in the interaction regions of the mean FA skeleton mask, with left corticospinal tract [*F* (1, 13) = 6.553, *P* = 0.024] and left inferior cerebellar peduncle [*F* (1, 13) = 7.590, *P* = 0.016] in the JHU atlas. Figure 4 illustrates the regression of mean FA values on PTSD severity scores for these two identified regions. No ROI showed a significant correlation between MD measures and PTSD severity.

### Connectome-based group-wise analysis

The connectome-based group-wise statistical analysis did not demonstrate any network or structural connection of DNUM or DFA that was significantly different between the PTSD and the trauma-exposed control group. As noted in the methods, multiple corrections were not applied for multiple testing.

## Discussion

The aim of this study was to explore the association between chronic PTSD, cortical atrophy, and WM microstructure among combat-exposed ADF veterans screened for potential confounding medical and psychiatric co-morbidities (including TBI, MDD, Alcohol Dependence, and current Antidepressant medication use). The volumetric analysis demonstrated that PTSD participants had significantly smaller volumes in the left PFC, bilateral middle frontal gyrus of the PFC, and left anterior insula compared with controls. This is consistent with previous literature that has established the PFC as an important region in PTSD brain pathophysiology and atrophy [5]. Previous research has also shown the middle frontal gyrus to be associated with the predictive contingency awareness between an aversive conditioned stimulus and unconditioned stimulus [47]. Finally, decreased bilateral insula volumes have been observed among survivors of fires who developed PTSD compared to non-PTSD survivors [48], and left insula volume has been observed to correlate with PTSD severity in a population of combat veterans [49] as well as those in the general population [50]. Finally, the anterior insula has been linked with negative emotional processing in PTSD and fear conditioning among healthy populations [6]. These findings are consistent with the neurobiological model of PTSD, which postulates that neural abnormalities in such regions may be implicated in the persistence of fear responses and emotion dysregulation characteristic of PTSD [2].

Results of this study indicated the PTSD group had significantly larger volumes of the left anterior orbital gyrus of the PFC than the control group. This is in contrast to the majority of literature that has demonstrated reduced, rather than increased volumes in those with PTSD [5]. However, some previous studies have also demonstrated this finding, with increased volume and cerebral blood flow in the orbitofrontal region observed among patients with PTSD and anxiety disorders [51, 52]. Activation of the orbitofrontal cortex has been associated with the retrieval of negative memories and anticipation of aversive stimuli, with larger volume associated with more severe symptoms of rumination in those with GAD [52].

When determining the association between PTSD severity and GM volume, a negative correlation was observed in the right central operculum. The operculum has been shown to have abnormal connectivity with the orbital frontal cortex in a previous study of PTSD [11]. Further, a positive correlation between PTSD severity and GM volume was observed in the right inferior occipital gyrus, a region that has been implicated in facial processing [53]. Previous fMRI research has shown activation of the occipital lobe among PTSD patients when in dissociative states following exposure to traumatic material [54]. However, given the low incidence of participants reporting dissociative symptoms in the PTSD group (*n* = 1), it is unlikely that dissociation accounted for this finding. Other regions typically implicated in studies of PTSD (hippocampus, and ACC) [5, 8, 12] did not demonstrate volumetric differences in or correlation with PTSD severity in this study. This is contrary to the hypotheses that hippocampal differences may impair contextual processing in those with PTSD, and differences in the ACC are linked to altered threat detection [2].

In terms of WM outcomes, no statistically significant variation in FA or MD values between PTSD participants and controls was found either globally or in ROI analyses using both TBSS and voxel-wise statistical methods. Connectome-based analysis also failed to discern differences between the PTSD and the control groups used in the study. This is in contrast to previous DTI studies which have found WM differences in the cingulum [11, 17, 22, 23], fronto-occipital fasciculus [21, 22], and other reported regions (i.e., thalamic radiations, inferior and superior longitudinal fasciculus, uncinate fasciculus, stria terminalis, and fornix) with conflicting directional findings [11, 17, 22].

It is possible the lack of detected differences in the current study may be related to power, given the small sample size of the PTSD group. However, it could also be indicative of the reduced impact of comorbidities, or the unique brain physiology of chronic PTSD sufferers which has been hypothesized to vary from acute PTSD sufferers in previous DTI research [16]. An alternative explanation for the lack of differences may be due to the matching of trauma exposure in both groups and the influence of trauma itself on WM microstructure. O’Doherty and colleagues [27] found no WM differences between PTSD sufferers and trauma-exposed controls in a sample of motor vehicle accident victims. However, the authors did find a number of differences in WM, including decreased FA, in both trauma-exposed participants with and without PTSD when compared with healthy non trauma-exposed controls.

Despite the lack of group-wise differences, analyses between PTSD severity scores and regional FA values in the PTSD group demonstrated significant negative correlations in the left corticospinal tract and left inferior cerebellar peduncle. Both of these tracts reportedly connect central networks involved in cognition and affect [14], and as PTSD symptoms increased within this sample, FA values in these areas decreased. Compromised cerebellar connectivity has been linked to emotional dysregulation, with tracts of the cerebellum observed to connect with the PFC [14, 55].

Given the role of the PFC in complex thought, problem solving and emotional processing, the findings extend the understanding of the psychiatric expression of PTSD and may contribute to assessing suitability of treatments in the future, in particular cognitive therapies. A high level of complex and abstract processing is required in cognitive therapies which are characterised by observing and identifying thinking patterns, determining the rationality of thoughts and beliefs, and generating new thought patterns in response to stimulus. These skills are developed and practiced over time with guidance by the therapist, and form an important component of TF CBT, the first line treatment for PTSD. While the efficacy of TF CBT is well-established, it has also been found that up to 50% of PTSD sufferers do not respond to first-line treatment, with chronic sufferers (like the current sample) notably more difficult to treat [56]. It may be possible that compromised WM microstructure in the PFC could contribute to the lack of response among this group in particular. However, further investigation into the link between treatment response and neuropathophysiology within the veteran population is needed. A recent systematic review on neurological changes following evidence-based trauma-focused therapies concluded that a potential mechanism of change in PTSD treatment may be an upregulation of the medial PFC, indicating that neural properties of this region may impact on the results or suitability of cognitive treatments [29].

In relation to previous research, Davenport and colleagues [22] found PTSD was associated with higher FA in a number of brain regions including the right corticospinal tract, bilateral middle cerebellar peduncle, and left superior cerebellar peduncle, while Hu and colleagues [12] found PTSD sufferers showed lower FA values in multiple regions of both hemispheres, including the corticospinal tract, compared with a trauma-exposed control group. This finding highlights the inconsistency within WM and PTSD literature related to directional restriction of diffusion. The current study contributes evidence that increased PTSD severity is related to reduced FA.

## Limitations and future directions

While this study has a number of unique strengths including the homogeneity of the sample and exclusion of confounding co-morbidities, there are limitations to consider when interpreting the results. First, the sample size of the PTSD group was small, compromising power to detect potential differences between the groups. This, along with discrepant findings in the literature, limited the ability to develop specific hypotheses and apply corrections for multiple testing. Second, there were relatively low levels of PTSD symptom severity within the PTSD group at the time of participation. The average CAPS-5 score in the PTSD group was 19.81 of a possible 80 total score, representing the lower end of severity. Further, while sub-threshold, 77% of the participants within the control group reported experiencing some symptoms of PTSD at the time of assessment. Although diagnostically distinctive, the groups may have been too similar in terms of trauma exposure and presence of PTSD symptoms to demonstrate meaningful WM differences. That said, lifetime (rather than current) PTSD diagnosis was considered more appropriate for investigating the impact of chronic and enduring PTSD symptoms, rather than acute PTSD on cortical volume and WM structural differences. This was also considered the most clinically appropriate method for examining neuroanatomical differences in a sample of veterans who had been exposed to combat more than 40 years prior. Furthermore, the groups were still distinguished by significantly different PTSD symptom severity.

Finally, the absence of a healthy, non-trauma-exposed control group prevents the ability to determine the true impact of trauma exposure in this sample, as we cannot detect potential differences attributable to trauma exposure alone. It is also possible the trauma-exposed control group may in fact be a particularly resilient group, given the absence of PTSD, mood, or substance use disorders late in life despite trauma exposure. It is important to consider whether neuroanatomical differences may be attributable to this group having pre-existing neuroanatomical features that serve as a protective predisposition.

Future DTI research among veterans should progress in a number of ways. A larger sample and comparison of three groups including PTSD (with moderate or above severity), trauma-exposed controls, and non trauma-exposed/healthy controls while retaining the stringent exclusion strategy in this study, will help delineate the impact of trauma and PTSD on WM microstructure among veterans with more clarity. Consideration of treatment history and response to first-line treatments in relation to symptom severity and WM properties would be valuable. Further, given the opposing directional findings of the association between FA and PTSD in the current literature, a meta-analysis of veteran DTI studies to date, may be of value.

## Conclusions

To the author’s knowledge, this is the first Australian study that has examined WM microstructural differences in veterans with PTSD. The study found CAPS-5 severity scores were negatively correlated with regional FA values in the left corticospinal tract and left inferior cerebellar peduncle in the PTSD group. The volumetric analysis also demonstrated that PTSD participants had significantly smaller volumes in the left PFC, bilateral middle frontal gyrus of the PFC, and left anterior insula, compared with controls. No WM differences were found between PTSD-diagnosed participants and trauma-exposed controls in the cingulum region, a commonly cited area of difference in other PTSD studies with military veterans. Further, no WM connectome-based group-wise differences between PTSD sufferers and trauma-exposed controls were observed in a sample free from confounding medical and psychiatric co-morbidities. The current study contributes to evidence that increased PTSD symptom severity is associated with the differences in WM structures indexed by decreased FA values.

## Data Availability

The data that support the findings of this study are available from Gallipoli Medical Research Foundation but restrictions apply to the availability of these data, which were used under license for the current study, and so are not publicly available. Data are however available from the authors upon reasonable request and with permission of the Australian Government Department of Veterans’ Affairs.

## Abbreviations

AAL: Automated anatomical labeling
ACC: Anterior cingulate cortex
ADF: Australian Defence Force
AUDIT: Alcohol Use Disorders Identification Test
CAPS-5: Clinician Administered PTSD Scale for DSM-5
CSF: Cerebrospinal fluid
DTI: Diffusion Tensor Imaging
DSM-5: Diagnostic and Statistical Manual of Mental Disorders
FA: Fractional anisotropy
FLAIR: Fluid attenuated inversion recovery
FMRIB: Functional MRI of the Brain
FSL: FMRIB software library
FWE: Family-wise error
GM: Grey matter
GMRF: Gallipoli Medical Research Foundation
GPH: Greenslopes Private Hospital
JHU: John Hopkins University
MANOVA: Multivariate analyses of variance
MD: Mean diffusivity
MDD: Major depressive disorder
MINI: Mini International Neuropsychiatric Interview
MoCA: Montreal Cognitive Assessment
MPRAGE: Magnetization-prepared rapid gradient-echo
MRI: Magnetic resonance imaging
NBS: Network-Based Statistics
PFC: Prefrontal cortex
PTSD: Posttraumatic stress disorder
ROI: Regions of interest
RSL: Returned and Services League
TBSS: Tract-based spatial statistics
TFCE: Threshold-free cluster enhancement
TF CBT: Trauma-focused cognitive behaviour therapy
TBI: Traumatic brain injury
WM: White matter
